# *TRAPPC11* and *GOSR2* mutations associate with hypoglycosylation of α-dystroglycan and muscular dystrophy

**DOI:** 10.1186/s13395-018-0163-0

**Published:** 2018-05-31

**Authors:** Austin A. Larson, Peter R. Baker, Miroslav P. Milev, Craig A. Press, Ronald J. Sokol, Mary O. Cox, Jacqueline K. Lekostaj, Aaron A. Stence, Aaron D. Bossler, Jennifer M. Mueller, Keshika Prematilake, Thierry Fotsing Tadjo, Charles A. Williams, Michael Sacher, Steven A. Moore

**Affiliations:** 1 Department of Pediatrics, University of Colorado School of Medicine and Children’s Hospital Colorado, Aurora, CO USA; 2 Department of Biology, Concordia University, Montreal, Canada; 3 Department of Pathology Carver College of Medicine, The University of Iowa, Iowa City, IA USA; 4 Division of Genetics and Metabolism, University of Florida College of Medicine, Gainesville, FL USA; 5 Department of Anatomy and Cell Biology, McGill University, Montreal, Canada

**Keywords:** TRAPPC11, GOSR2, Golgi, Dystroglycanopathy, Dystroglycan, Muscular dystrophy, Glycosylation, Membrane traffic

## Abstract

**Background:**

Transport protein particle (TRAPP) is a supramolecular protein complex that functions in localizing proteins to the Golgi compartment. The TRAPPC11 subunit has been implicated in muscle disease by virtue of homozygous and compound heterozygous deleterious mutations being identified in individuals with limb girdle muscular dystrophy and congenital muscular dystrophy. It remains unclear how this protein leads to muscle disease. Furthermore, a role for this protein, or any other membrane trafficking protein, in the etiology of the dystroglycanopathy group of muscular dystrophies has yet to be found. Here, using a multidisciplinary approach including genetics, immunofluorescence, western blotting, and live cell analysis, we implicate both TRAPPC11 and another membrane trafficking protein, GOSR2, in α-dystroglycan hypoglycosylation.

**Case presentation:**

Subject 1 presented with severe epileptic episodes and subsequent developmental deterioration. Upon clinical evaluation she was found to have brain, eye, and liver abnormalities. Her serum aminotransferases and creatine kinase were abnormally high. Subjects 2 and 3 are siblings from a family unrelated to subject 1. Both siblings displayed hypotonia, muscle weakness, low muscle bulk, and elevated creatine kinase levels. Subject 3 also developed a seizure disorder. Muscle biopsies from subjects 1 and 3 were severely dystrophic with abnormal immunofluorescence and western blotting indicative of α-dystroglycan hypoglycosylation. Compound heterozygous mutations in *TRAPPC11* were identified in subject 1: c.851A>C and c.965+5G>T. Cellular biological analyses on fibroblasts confirmed abnormal membrane trafficking. Subject 3 was found to have compound heterozygous mutations in *GOSR2*: c.430G>T and c.2T>G. Cellular biological analyses on fibroblasts from subject 3 using two different model cargo proteins did not reveal defects in protein transport. No mutations were found in any of the genes currently known to cause dystroglycanopathy in either individual.

**Conclusion:**

Recessive mutations in *TRAPPC11* and *GOSR2* are associated with congenital muscular dystrophy and hypoglycosylation of α-dystroglycan. This is the first report linking membrane trafficking proteins to dystroglycanopathy and suggests that these genes should be considered in the diagnostic evaluation of patients with congenital muscular dystrophy and dystroglycanopathy.

## Background

Dystroglycanopathies are a group of muscular dystrophies resulting from abnormal glycosylation of α-dystroglycan (α-DG) that leads to reduced binding affinity for extracellular matrix proteins [1]. The clinical phenotypes span a broad range from the congenital muscular dystrophies (CMDs) with brain and eye malformations to adult-onset limb-girdle muscular dystrophy (LGMD) [2]. Dystroglycan is encoded by *DAG1* and is cleaved into α-DG and β-DG after translation [3]. *DAG1* is widely expressed in different human tissues, consistent with the multi-organ phenotypes of many individuals with the most severe forms of dystroglycanopathy [4].

Mutations in *DAG1* itself as well as 17 other genes have been reported in patients with dystroglycanopathy. These include glycosyltransferases (*POMT1*, *POMT2*, *POMGNT1*, *POMGNT2*, *B3GALNT2*, *B3GNT1*, *LARGE*, *TMEM5*), a kinase (*POMK*), five genes encoding enzymes necessary for dolichol-P-mannose (dol-P-man) synthesis (*DOLK*, *DPM1*, *DPM2*, *DPM3*, and *GMPPB*), and three genes encoding proteins necessary for joining the α-DG-linked core glycan structure with the distal ligand-binding region of the structure via a ribitol phosphate disaccharide (*FKTN*, *FKRP*, *ISPD*) [5]. To date, no membrane trafficking proteins have been implicated in dystroglycanopathies.

In this study, we report clinical, histopathological, biochemical, and molecular genetic data on two families with CMD and hypoglycosylation of α-DG. Two genes, *TRAPPC11* and *GOSR2*, that each have a role in membrane trafficking in the biosynthetic pathway have been implicated as candidate dystroglycanopathy genes. They represent the first membrane trafficking proteins implicated in α-DG hypoglycosylation. Since *TRAPPC11* mutations have been reported in a number of individuals suffering from a muscular dystrophy, and these individuals also display membrane trafficking defects in cultured fibroblasts, this gene should be considered in the diagnostic evaluation of patients with CMD.

## Case presentation

### Family 1

**Fig. 1:**
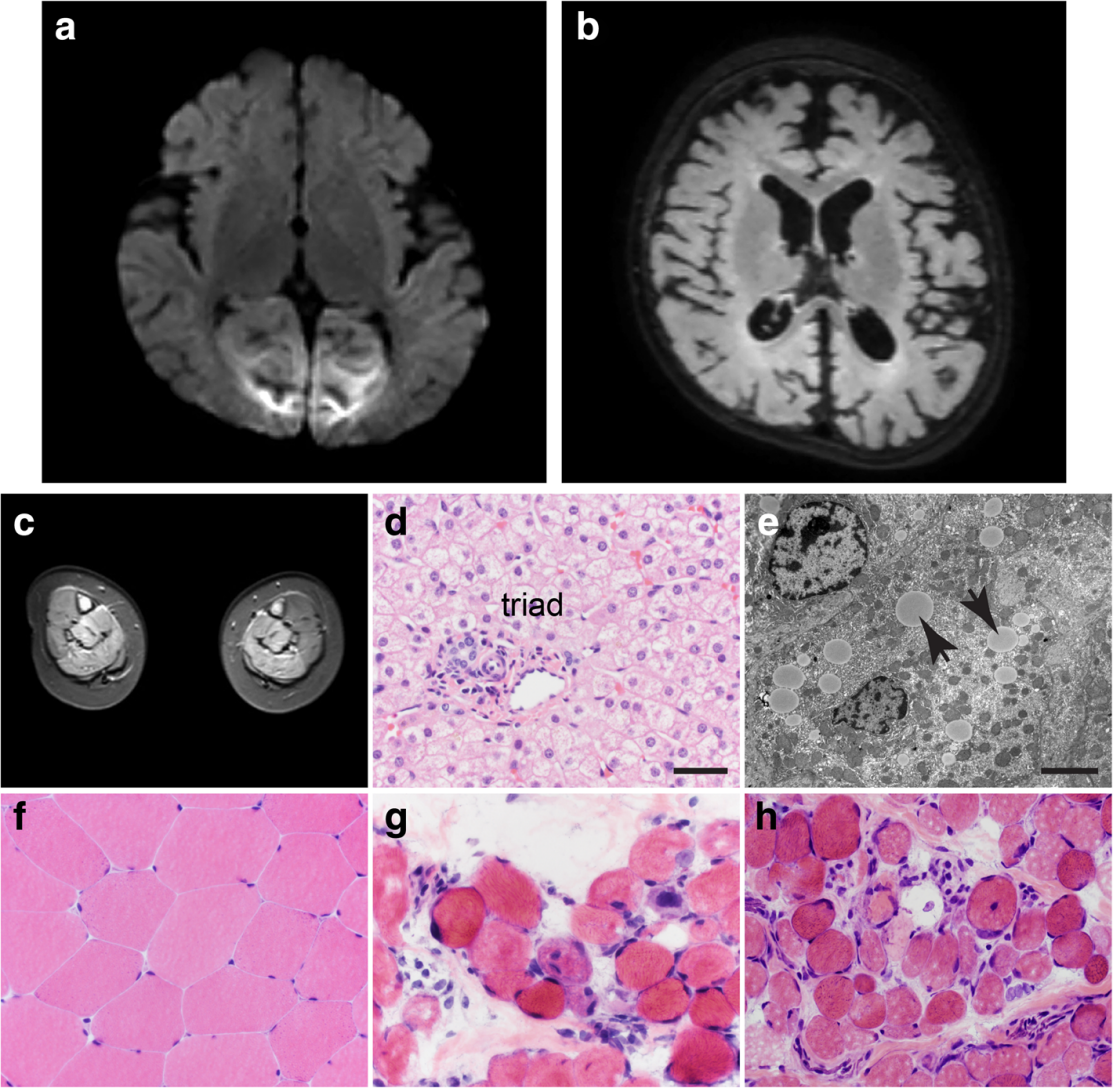
Subjects 1 and 3 display brain, liver, and muscle abnormalities. **a** Diffusion-weighted (B1000) MRI showing restricted diffusion of the medial occipital cortex and underlying white matter at 6 months of age in subject 1 at the time of initial presentation. **b** Fluid-attenuated inversion recovery (FLAIR) MRI for subject 1 at 15 months notable for marked cerebral volume loss. **c** Short tau inversion recovery (STIR) shows symmetric high signal in the posterior compartments of the legs of subject 1 at 12 months of age. Subject 1 has microvesicular steatosis of the liver; light microscopy hematoxylin and eosin (**d**) and electron microscopy (**e**). Note the lipid accumulations marked by the arrows in **e**. **f**–**h** Muscle biopsies from control (**f**), subject 1 (**g**), and subject 3 (**h**) were stained with hematoxylin and eosin. Dystrophic features are present in subjects 1 and 3. The size bar denotes 50 μm in **d** and **f**–**h**. The size bar denotes 5 μm in **e**

Subject 1 presented with status epilepticus in the setting of a vomiting illness at 6 months of age. Magnetic resonance imaging (MRI) of the brain showed bilateral multifocal restricted diffusion of the cortex, the cerebral white matter, and the pons (Fig. 1a). Her serum aminotransferases were elevated with alanine aminotransferase (ALT) of ~ 1600 U/L and aspartate aminotransferase (AST) ~ 400 U/L as well as a prolonged prothrombin time of 20.7 s (normal range is 12–15 s), consistent with synthetic liver dysfunction. The approximately 4:1 ALT to AST ratio was consistent over multiple measurements. Creatine kinase (CK) at initial presentation was 3500 U/L. She had significant regression of development with loss of rolling and sitting, loss of fine motor and verbal skills, and inability to feed orally after this illness.

**Fig. 2:**
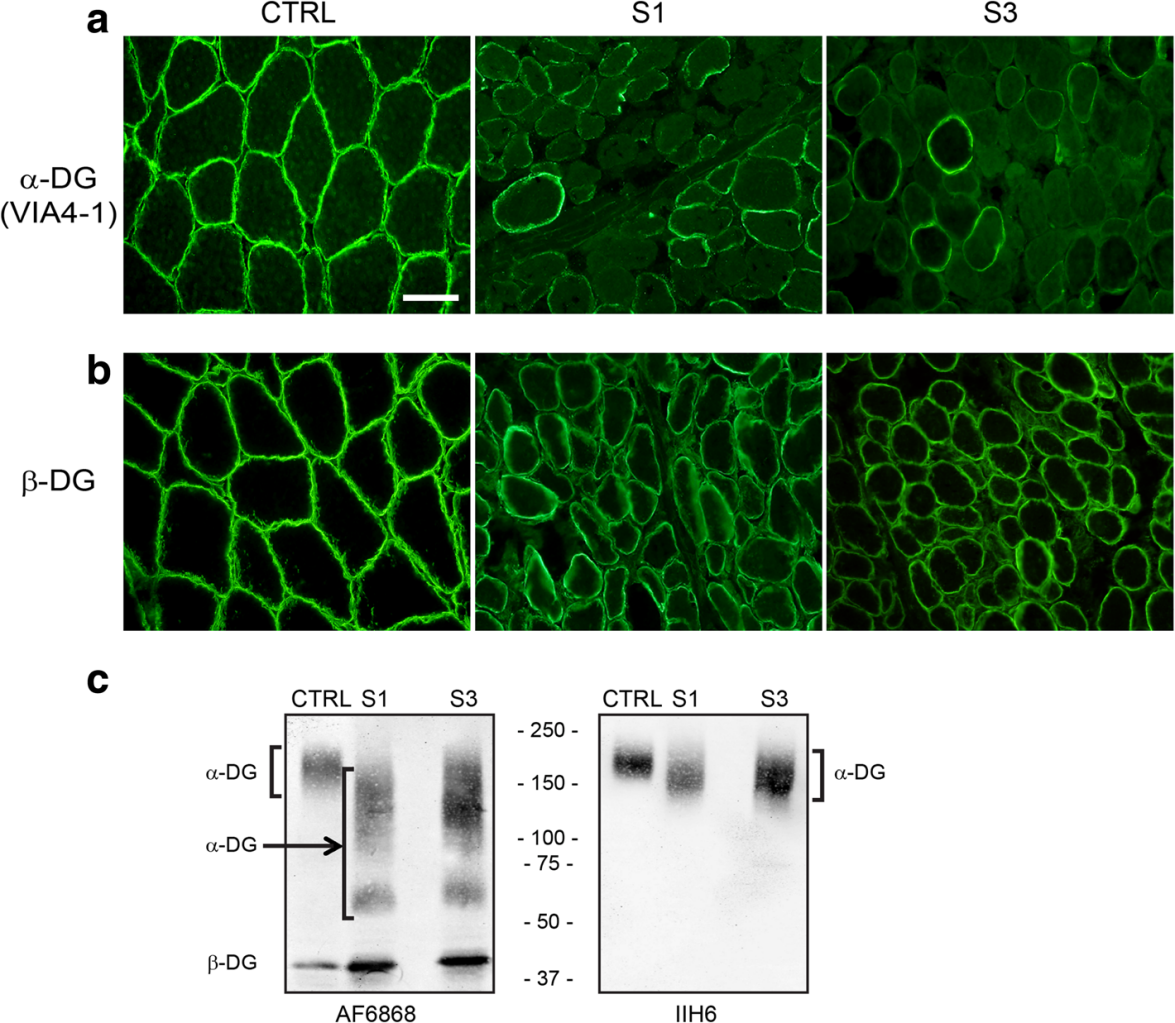
Subjects 1 and 2 display abnormalities in both α-dystroglycan staining and glycosylation. Control muscle or muscle taken from subject 1 (S1) and subject 3 (S3) were stained for alpha dystroglycan using VIA4-1 antibody (**a**) or β-DG (**b**). Note the reduced staining for α-DG but not β-DG in subjects 1 and 3. The size bar denotes 50 μm for all panels in **a** and **b**. **c** Western blot analysis of muscle tissue from control and subjects 1 and 3. Samples were probed with peptide-specific antibody AF6868 and the glycoepitope-specific antibody IIH6 as indicated. The location of α-DG and β-DG is indicated. Note that control shows a higher molecular size immunoreactive species for α-DG with both antibodies while S1 and S3 show a more heterogeneous species of much smaller molecular size, suggesting hypoglycosylation of the protein

MRI of the lower extremities showed high signal on short tau inversion recovery (STIR) sequences of the deep and superficial posterior compartments bilaterally (Fig. 1c). Skeletal muscle and liver biopsies were obtained at 9 months of age. Liver biopsy showed microvesicular steatosis (Fig. 1d, e). Skeletal muscle showed an active dystrophic process (Fig. 1g) and hypoglycosylation of α-DG by both immunofluorescence and western blotting (Fig. 2). In contrast, α-DG in cultured fibroblasts was indistinguishable from control fibroblasts in on-cell and in WGA glycoprotein western blots (data not shown). This is not uncommon and has been reported in the case of other genes involved in dystroglycanopathy [6, 7].

The patient had several subsequent acute infectious illnesses with seizures and neurological regression. Follow-up brain MRI at 15 months of age showed marked progressive volume loss (Fig. 1b). Glycosylation analysis of transferrin and ApoCIII proteins in serum by affinity chromatography-mass spectrometry (Mayo Medical Laboratory) as well as by MALDI-TOF mass spectrometry (Emory Genetics Laboratory) was normal. In her last evaluation, at 3 years and 6 months of age, she was areflexic with limited antigravity strength and severe hypotonia. She fed exclusively via gastrostomy tube with no verbal communication. Seizures were well controlled on levetiracetam monotherapy. CK remained elevated with values as high as 19,000 U/L. She had mild hepatomegaly and aminotransferases were still significantly elevated with 4:1 ALT to AST ratio, but there was no coagulopathy or hyperbilirubinemia. She chronically required noninvasive positive pressure ventilation with sleep. After multiple hospital admissions for viral respiratory infections, she underwent immunological evaluation and was found to have impaired natural killer cell function on multiple repeated analyses. She did not have peripheral neuropathy, cataracts, alacrima, achalasia, renal disease, hearing loss, or cholestasis.

**Fig. 3:**
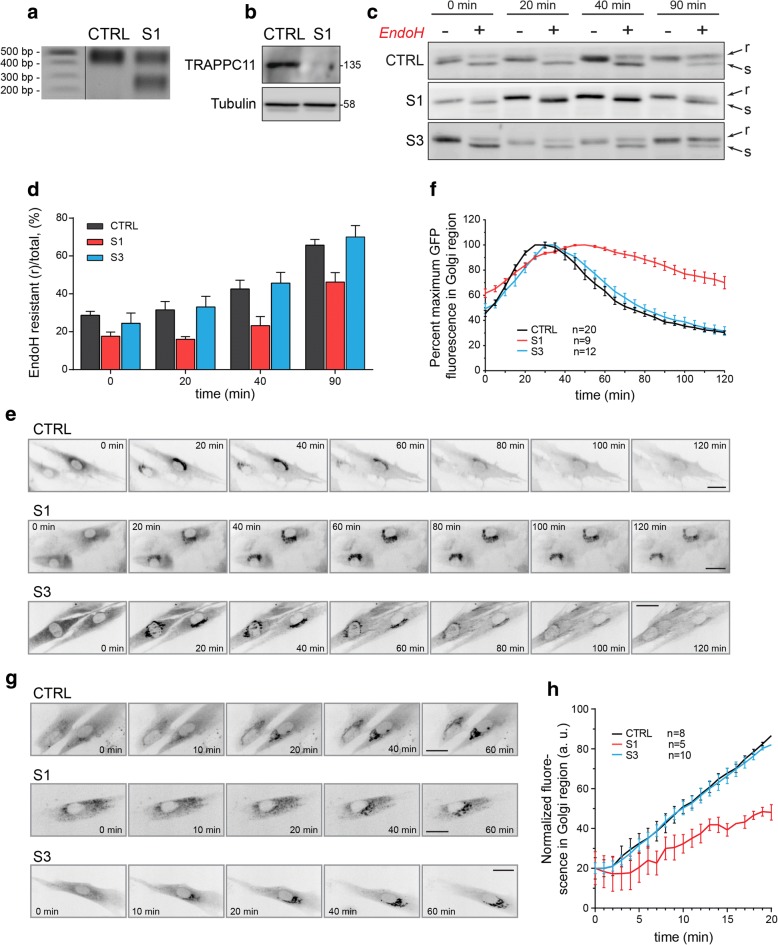
*TRAPPC11* compound heterozygous mutations affect membrane trafficking in patient fibroblasts. **a** mRNA was collected from control and subject 1 (S1), converted to cDNA and amplified by PCR using oligonucleotides annealing to exons 8 and 11. The amplicons were sequenced and found to represent exons 8-9-10-11 (higher molecular size amplicon) and exons 8-part of 10-11 (lower molecular size amplicon). **b** Lysates from control and subject 1 (S1) fibroblasts were probed for TRAPPC11 and tubulin as a loading control. **c** Fibroblasts were infected with VSVG-GFP ts045, and the protein was arrested in the endoplasmic reticulum (ER) by shifting the cells to 40 °C. The protein was synchronously released from the ER upon downshifting the temperature to 32 °C, and the acquisition of Endoglycosidase H (EndoH) resistance was assayed at the times indicated. A representative western blot is displayed, and quantification of a minimum of three such blots is shown in **d**. **e** The same assay as in **b** was performed on live cells and the arrival and release of the GFP signal was quantified over time. Representative images from the movies are displayed in **e**, and quantification of the signal in the Golgi region is shown in **f**. To more accurately measure ER-to-Golgi trafficking, the RUSH assay [36] was performed using ST-SBP-GFP with the Ii hook (**g**). Images were acquired over time in live cells upon addition of biotin to initiate release of the protein from the ER. Quantification of the signal in the Golgi is displayed in **h**. Size bars in **e** and **g** denote 25 μm. Error bars represent SEM from a minimum of three replicates in **d**. *N* values for **f** and **h** are indicated in the figure

Exome trio sequencing showed compound heterozygous rare variants *in trans* in *TRAPPC11* (NM_021942): c.851A>C (p.Q284P) and c.965+5G>T (intron 9 splice site disruptor). The p.Q284P missense mutation was absent from the Exome Aggregation Consortium (ExAC) database, and c.965+5G>T was present in 2/119,770 alleles [8]. The latter mutation resulted in a transcript that lacks exon 9 and the first 88 bases of exon 10 (Fig. 3a) and is predicted to result in an in-frame deletion of amino acids 278–351 (p.I278_Q351del). Cultured fibroblasts had greatly reduced levels of TRAPPC11 (Fig. 3b) suggesting the p.Q284P protein and the predicted p.I278_Q351del protein are unstable. These fibroblasts showed a delay in the maturation of the marker protein VSVG-GFP ts045 (Fig. 3c, d). Analysis of live-cell trafficking revealed a delay in the release of VSVG-GFP ts045 from the Golgi (Fig. 3e, f) as well as a delay in arrival of a Golgi marker (sialyl transferase-SBP-GFP) from the endoplasmic reticulum (Fig. 3g, h). The delayed release of protein from the Golgi is consistent with the initial findings reported by Bögershausen et al. in LGMD2S patients with *TRAPPC11* mutations [9], and the delayed arrival of protein to the Golgi is consistent with the findings of Scrivens et al. [10].

### Family 2

Subject 2 presented for medical evaluation at age 6 months for hypotonia. She was found to have CK values of up to ~ 5000 U/L. She developed absence seizures at age 2 years. She had steadily progressive muscle weakness. On examination at age 4 years and 9 months, she was areflexic. She had low muscle bulk and myopathic facial appearance and did not have antigravity strength. She had fine nystagmus but otherwise intact extraocular movements. MRI of the brain was normal at ages 2 and 4 years. She died due to respiratory failure at age 5 years.

Subject 3 is the younger sister of subject 2. She was noted to have muscle weakness and hypotonia at 3 months of age. At 9 months, she had antigravity strength only. Serum CK value was 1760 U/L. At 19 months, skeletal muscle biopsy was obtained showing an active dystrophic process (Fig. 1h) and hypoglycosylation of α-DG by both immunofluorescence and western blotting (Fig. 2). In contrast, α-DG in cultured fibroblasts was indistinguishable from control fibroblasts in on-cell and in WGA glycoprotein western blots (data not shown). Furthermore, the VSVG-GFP membrane trafficking assay kinetics as well as arrival of the Golgi marker was indistinguishable from control fibroblasts (Fig. 3c–f).

At age 2.5 years, she developed a seizure disorder characterized as focal seizures but later as both focal and generalized, which often became intractable and required hospitalization. Evaluation showed no evidence of nystagmus and ocular range of motion was full. There were no focal deficits and her cranial nerves were normal. She demonstrated severe weakness and muscular hypotonia. MRI of the brain showed diffuse volume loss resulting in *ex vacuo* ventriculomegaly. EEG at 2 years and 7 months of age showed runs of spike and wave discharges originating in the occipital lobe which were exacerbated by photic stimuli. Head circumference was at the 30th centile, height at the 10th centile, and weight below the 1st centile.

At 3.5 years of age, she developed episodes of vomiting and apparent abdominal pain. This led to the detection of elevated ALT of up to 700 U/L. An extensive evaluation for infectious, anatomical, autoimmune, and metabolic etiologies of liver disease was nondiagnostic.

Physical exam revealed findings similar to her sister. Apart from her sister, there is no family history of neuromuscular disease. The subject is now 6 years of age with medically refractory epilepsy and progressive severe muscle weakness. Clinical exome trio sequencing was performed, and no relevant sequence variants were initially reported. In a targeted sequencing panel, subject 3 was found to have compound heterozygous rare variants in *GOSR2* (NM_001012511): c.430G>T (p.G144W) and c.2T>G. Retrospective evaluation of *GOSR2* in the whole exome sequencing (WES) data confirmed that both variants were present in subject 3 and were *in trans*. Extensive re-evaluation of seizure and dystroglycanopathy loci in the WES failed to identify any other pathologic variants. The GOSR2 p.G144W missense variant is a previously reported disease-causing mutation and is present in 5/121,408 alleles in the ExAC database with no homozygous individuals. The second variant (c.2T>G) is present in 1/18,808 alleles in the ExAC database [8]. The mutation is likely to result in the use of an alternate start codon with elimination of 18 amino acids from the amino-terminus of the protein according to MutationTaster2 and is presumed to be pathogenic as a result [11].

## Discussion and conclusions

In this report, we show that mutations in two genes encoding proteins involved in membrane trafficking, *TRAPPC11* and *GOSR2*, are associated with CMD and dystroglycanopathy. Biallelic mutations in *TRAPPC11* were initially reported as the etiology of LGMD2S in 2013 [9] and have since been associated with a variety of multisystemic phenotypic findings including intellectual disability, seizures, microcephaly, cerebral atrophy, cataracts, alacrima, achalasia, hepatic steatosis, and cholestatic liver disease, in addition to muscular dystrophy [9, 12–15]. Comparisons between subject 1 and all published mutations in *TRAPPC11* and associated phenotypes are summarized in Table 1. Our study now adds two new mutations with functional validation and categorizes *TRAPPC11*-related disease as a dystroglycanopathy.

**Table 1:** Comparison of all known TRAPPC11 and GOSR2 mutations

Genotype	Number of cases	Neurological phenotype	Muscle phenotype	Other features	References
*TRAPPC11* c.2938G>A c.2938G>A	3	motor delay in one individual, otherwise normal	LGMD, CK up to ~ 2800	scoliosis, cataracts and esotropia each in one individual	Bogershausen et al. [9]
*TRAPPC11* c.1287+5G>A c.1287+5G>A	5	epilepsy, developmental delay, ataxia, chorea, microcephaly, cerebral atrophy	myopathy, CK up to ~ 1200	short stature, exophoria in one individual	Bogershausen et al. [9]
*TRAPPC11* c.1893+3A>G c.1893+3A>G	4	developmental delay, cerebral atrophy, medically refractory epilepsy	CMD, CK not reported, dystrophic appearance of biopsied muscle tissue	scoliosis, achalasia, alacrima	Koehler et al. [14]
*TRAPPC11* c.513_516delTTTG c.2330A>C	2	moderate intellectual disability, ambulatory, seizures, MRI with mild atrophy	CMD, CK up to ~ 10,000; dystrophic biopsied muscle, abnormal dystroglycan staining	cataracts, significantly elevated ALT, mildly elevated AST, liver fibrosis	Fee et al. [15]
*TRAPPC11* c.2938G>A c.661-1G>T	1	developmental delay, decreased white matter volume on MRI	CMD, CK up to ~ 9000; abnormal signal in posterior compartment leg muscles on CT scan	hepatic steatosis, significantly elevated ALT, mildly elevated AST, cataracts	Liang et al. [13]
*TRAPPC11* c.1141C>G c.3310A>G	1	microcephaly, brain atrophy on MRI, sensorineural hearing loss, peripheral neuropathy	presumed CMD, hypotonia, CK not reported	retrognathia, cholestatic liver disease, thrombocytopenia, nephropathy, osteopenia	Matalonga et al. [12]
*TRAPPC11* c.851A>C c.965+5G>T	1	severe developmental delay, multifocal restricted diffusion on MRI; later cerebral atrophy	CMD, CK up to ~ 18,000; abnormal signal in posterior compartment leg muscles on MRI scan; dystrophic appearance of biopsied muscle; hypoglycosylation of α-dystroglycan	hepatic steatosis, significantly elevated ALT, mildly elevated AST; retinopathy, impaired NK cell function	This paper
*GOSR2* c.430G>T c.430G>T	17	“North Sea” progressive myoclonus epilepsy; childhood-onset ataxia, loss of ambulation in early adulthood	CK up to ~ 2500 but normal in some; no specific abnormalities reported in muscle biopsies	scoliosis, pes cavus, syndactyly in some, delayed puberty in some	Lomax et al. [19], Egmond et al. [20], Corbett et al. [18]
*GOSR2* c.430G>T c.491_493delAGA	1	progressive myoclonus epilepsy, ataxia; MRI with cerebral atrophy	none reported	none reported	Praschberger et al. [37]
*GOSR2* c.430G>T c.2T>G	2	medically refractory epilepsy; MRI with cerebral atrophy	CMD, CK up to ~ 5000; dystrophic muscle biopsy with hypoglycosylation of α-dystroglycan; severe weakness and respiratory failure leading to death at 5 years in older sibling	no additional findings	This paper

TRAPPC11 dysfunction may contribute to disease pathophysiology in several ways. Extensive functional studies of cultured fibroblasts were conducted by Bögershausen et al. [9]. They demonstrated that cells had abnormally fragmented and diffuse Golgi; delayed traffic out of the Golgi and the proteins LAMP1 and LAMP2 were found to be abnormally glycosylated. TRAPP (transport protein particle) forms several related multisubunit trafficking complexes (MTCs) that participate in the tethering of vesicles to target membranes, including vesicles associated with the Golgi [10]. Since the Golgi is the major site of protein glycosylation in the cell [16], defects in Golgi morphology and traffic can result in protein glycosylation defects. Recently, abnormal glycosylation of serum transferrin was described in a patient with compound heterozygous mutations in *TRAPPC11*, consistent with a type 2 disorder of glycosylation [12]. We were unable to detect abnormalities in glycosylation of serum transferrin using two different commonly employed methods. Thus, while *TRAPPC11*-related disease is a disorder of glycosylation, analysis of glycoepitopes of secreted proteins may not be a sensitive test for diagnostic purposes.

The zebrafish model of *TRAPPC11*-related disease shows generalized impairment of *N*-linked glycosylation as well as depletion of lipid-linked oligosaccharides (LLOs) [17]. The inability to synthesize dolichol-P-mannose (dol-P-man), a lipid-linked saccharide, is a known cause of dystroglycanopathy [7]. Expression of multiple glycosylation-related genes (including the known etiologies of dystroglycanopathy *gmppb*, *dpm1*, *dpm2*, and *dpm3*) showed significant compensatory upregulation in the *trappc11* fish [17]. Interestingly, *TRAPPC11* siRNA knockdown in HeLa cells had a specific inhibitory effect on glycosylation that was not present with knockdown of other components of the TRAPP complex. This led to the conclusion that TRAPPC11 may have another function that is independent of its role in vesicle transport and led to speculation that impaired LLO synthesis may be the most relevant function of TRAPPC11 in the process of protein glycosylation [17]. Finally, *trappc11* zebrafish mutations were shown to lead to fatty liver via a pathological activation of the unfolded protein response. This may be relevant to subject 1 as well as the other reported individuals with hepatopathy and *TRAPPC11*-related disease [13]. Taken together, several mechanisms for the role of *TRAPPC11* in muscular and hepatic phenotypes are known and can explain many clinical features of subject 1.

Human mutations in *GOSR2* were first reported in 2011 in six individuals with the same homozygous missense mutation (c.430G>T) who had progressive myoclonus epilepsy (PME), ataxia, scoliosis, and mildly elevated serum CK (see Table 1 for a comparison between subjects 2 and 3 with all reported *GOSR2* mutations) [18]. All individuals were areflexic in early childhood and were non-ambulatory by adolescence or early adulthood. Muscle histology and EMG were normal. An additional eleven individuals with similar clinical presentations and the same homozygous mutation were reported in 2013 and 2014 [19, 20]. The maximum CK value reported in any of the patients was 2467 U/L. There was no specific assessment of α-DG glycosylation in their muscle biopsies. Subjects 2 and 3 in our study have a much more severe phenotype. Since CMD represents the severe end of the clinical spectrum of *GOSR2*-related disease and PME represents the milder end of the spectrum, the new c.2T>G mutation resulting in CMD reported in our study likely cause more severe perturbation of Golgi function than the common c.430G>T mutation. It remains unclear which aspect of Golgi function is affected since a membrane trafficking defect in neither the VSVG-GFP marker protein nor a resident Golgi enzyme was detected. Future studies should examine the trafficking of Golgi-localized glycosyl transferases that are responsible for α-DG processing.

*GOSR2* encodes a Golgi Qb-SNARE (soluble *N*-ethylmaleimide-sensitive factor attachment protein receptor) protein. In the cell, GOSR2 localizes to the *cis*-Golgi and mediates docking and fusion of vesicles originating from the ER. There is precedent for Golgi dysfunction leading to diseases manifesting with abnormal glycosylation and multisystemic disease. Examples include the disease caused by mutations in genes that encode the COG (conserved oligomeric Golgi) complex, an MTC that localizes to the Golgi [21]. Additionally, an individual has been described with CMD due to homozygous mutations in *GOLGA2*, a golgin protein that also impacts Golgi function [22]. The potential for a link between aberrant Golgi trafficking and dystroglycanopathy stems from an experiment employing a modified virus that required normally glycosylated α-DG for cell entry. Knockouts of known dystroglycanopathy genes in cultured fibroblasts resulted in impaired viral cell entry. Among the other knockouts shown to impair viral cell entry were those cells with mutations in several of the COG complex genes [23].

Dystroglycanopathies result in muscular dystrophy due to dysfunctional linkage of the sarcolemma to the extracellular matrix. This linkage occurs via α-DG and relies on the synthesis of a complex LARGE-glycan for normal function [5]. Since the initial descriptions of dystroglycanopathy [1, 24–26], a variety of molecular mechanisms of the diseases have been discovered. Specific glycosyltransferases such as *POMT1/POMT2* are required to construct the core glycan structure that is linked to α-DG [25, 26]. Mutations in *DOLK*, *DPM1*, *DPM2*, *DPM3*, and *GMPPB* likely lead to a deficiency of dol-P-man (a lipid-linked monosaccharide) resulting in abnormal *N*-linked glycosylation as well as the *O*-linked mannosylation defect that results in dystroglycanopathy [6, 7, 27–31]. LARGE synthesizes the extracellular matrix binding region of the glycan structure (matriglycan) that is distal to the core region [32]. Most recently, FKTN, FKRP, and ISPD have been implicated in the addition of ribitol phosphate molecules to link the core and ligand-binding regions of the α-DG glycan structure [33–35]. Our study suggests that TRAPPC11 and GOSR2 are also involved in the trafficking and glycosylation of dystroglycan in the Golgi. This represents the first report of an association between these genes and α-DG hypoglycosylation. It remains to be seen if other *GOSR2* mutations associate with similar cellular and clinical phenotypes. Given the number of individuals with *TRAPPC11* mutation-associated muscular dystrophy, it may be prudent for this gene to now be considered in the diagnostic evaluation of patients with dystroglycanopathy.

## Abbreviations

ALT: Alanine aminotransferase
AST: Aspartate aminotransferase
CK: Creatine kinase
FLAIR: Fluid-attenuated inversion recovery
MRI: Magnetic resonance imaging
STIR: Short tau inversion recovery
α-DG: α-Dystroglycan

## References

[1] Michele DE, Barresi R, Kanagawa M, Saito F, Cohn RD, Satz JS, Dollar J, Nishino I, Kelley RI, Somer H (2002). Post-translational disruption of dystroglycan–ligand interactions in congenital muscular dystrophies. Nature..

[2] Bönnemann CG, Wang CH, Quijano-Roy S, Deconinck N, Bertini E, Ferreiro A, Muntoni F, Sewry C, Béroud C, Mathews KD (2014). Diagnostic approach to the congenital muscular dystrophies. Neuromuscul Disord..

[3] Ibraghimov-Beskrovnaya O, Milatovich A, Ozcelik T, Yang B, Koepnick K, Francke U, Campbell KP (1993). Human dystroglycan: skeletal muscle cDNA, genomic structure, origin of tissue specific isoforms and chromosomal localization. Hum Mol Genet..

[4] Uhlén M, Fagerberg L, Hallström BM, Lindskog C, Oksvold P, Mardinoglu A, Sivertsson Å, Kampf C, Sjöstedt E, Asplund A (2015). Proteomics. Tissue-based map of the human proteome. Science.

[5] Sheikh MO, Halmo SM, Wells L (2017). Recent advancements in understanding mammalian O-mannosylation. Glycobiology..

[6] Jensen BS, Willer T, Saade DN, Cox MO, Mozaffar T, Scavina M, Stefans VA, Winder TL, Campbell KP, Moore SA (2015). GMPPB-associated dystroglycanopathy: emerging common variants with phenotype correlation. Hum Mutat..

[7] Carss KJ, Stevens E, Foley AR, Cirak S, Riemersma M, Torelli S, Hoischen A, Willer T, van Scherpenzeel M, Moore SA (2013). Mutations in GDP-mannose pyrophosphorylase B cause congenital and limb-girdle muscular dystrophies associated with hypoglycosylation of α-dystroglycan. Am J Hum Genet..

[8] Lek M, Karczewski KJ, Minikel EV, Samocha KE, Banks E, Fennell T, O'Donnell-Luria AH, Ware JS, Hill AJ, Cummings BB (2016). Analysis of protein-coding genetic variation in 60,706 humans. Nature..

[9] Bögershausen N, Shahrzad N, Chong JX, von Kleist-Retzow J-C, Stanga D, Li Y, Bernier FP, Loucks CM, Wirth R, Puffenberger EG (2013). Recessive TRAPPC11 mutations cause a disease spectrum of limb girdle muscular dystrophy and myopathy with movement disorder and intellectual disability. Am J Hum Genet..

[10] Scrivens PJ, Noueihed B, Shahrzad N, Hul S, Brunet S, Sacher M (2011). C4orf41 and TTC-15 are mammalian TRAPP components with a role at an early stage in ER-to-Golgi trafficking. Mol Biol Cell..

[11] Schwarz JM, Cooper DN, Schuelke M, Seelow D (2014). MutationTaster2: mutation prediction for the deep-sequencing age. Nat Methods..

[12] Matalonga L, Bravo M, Serra-Peinado C, García-Pelegrí E, Ugarteburu O, Vidal S, Llambrich M, Quintana E, Fuster-Jorge P, Gonzalez-Bravo MN (2017). Mutations in TRAPPC11 are associated with a congenital disorder of glycosylation. Hum Mutat..

[13] Liang W-C, Zhu W, Mitsuhashi S, Noguchi S, Sacher M, Ogawa M, Shih H-H, Jong Y-J, Nishino I (2015). Congenital muscular dystrophy with fatty liver and infantile-onset cataract caused by TRAPPC11 mutations: broadening of the phenotype. Skelet Muscle.

[14] Koehler K, Milev MP, Prematilake K, Reschke F, Kutzner S, Jühlen R, Landgraf D, Utine E, Hazan F, Diniz G (2016). A novel TRAPPC11 mutation in two Turkish families associated with cerebral atrophy, global retardation, scoliosis, achalasia and alacrima. J Med Genet..

[15] Fee DB, Harmelink M, Monrad P, Pyzik E (2017). Siblings with mutations in TRAPPC11 presenting with limb-girdle muscular dystrophy 2S. J Clin Neuromuscul Dis..

[16] Ohtsubo K, Marth JD (2006). Glycosylation in cellular mechanisms of health and disease. Cell..

[17] DeRossi C, Vacaru A, Rafiq R, Cinaroglu A, Imrie D, Nayar S, Baryshnikova A, Milev MP, Stanga D, Kadakia D (2016). trappc11 is required for protein glycosylation in zebrafish and humans. Mol Biol Cell.

[18] Corbett MA, Schwake M, Bahlo M, Dibbens LM, Lin M, Gandolfo LC, Vears DF, O'Sullivan JD, Robertson T, Bayly MA (2011). A mutation in the Golgi Qb-SNARE gene GOSR2 causes progressive myoclonus epilepsy with early ataxia. Am J Hum Genet..

[19] Lomax LB, Bayly MA, Hjalgrim H, Møller RS, Vlaar AM, Aaberg KM, Marquardt I, Gandolfo LC, Willemsen M, Kamsteeg E-J (2013). ‘North Sea’progressive myoclonus epilepsy: phenotype of subjects with GOSR2 mutation. Brain..

[20] Egmond ME, Verschuuren-Bemelmans CC, Nibbeling EA, Elting JWJ, Sival DA, Brouwer OF, Vries JJ, Kremer HP, Sinke RJ, Tijssen MA (2014). Ramsay Hunt syndrome: clinical characterization of progressive myoclonus ataxia caused by GOSR2 mutation. Mov Disord..

[21] Freeze HH, Ng BG (2011). Golgi glycosylation and human inherited diseases. Cold Spring Harb Perspect Biol..

[22] Shamseldin HE, Bennett AH, Alfadhel M, Gupta V, Alkuraya FS (2016). GOLGA2, encoding a master regulator of golgi apparatus, is mutated in a patient with a neuromuscular disorder. Hum Genet..

[23] Jae LT, Raaben M, Riemersma M, van Beusekom E, Blomen VA, Velds A, Kerkhoven RM, Carette JE, Topaloglu H, Meinecke P (2013). Deciphering the glycosylome of dystroglycanopathies using haploid screens for lassa virus entry. Science..

[24] Yoshida A, Kobayashi K, Manya H, Taniguchi K, Kano H, Mizuno M, Inazu T, Mitsuhashi H, Takahashi S, Takeuchi M (2001). Muscular dystrophy and neuronal migration disorder caused by mutations in a glycosyltransferase, POMGnT1. Dev Cell..

[25] van Reeuwijk J, Janssen M, van den Elzen C, de Bernabe DB-V, Sabatelli P, Merlini L, Boon M, Scheffer H, Brockington M, Muntoni F (2005). POMT2 mutations cause α-dystroglycan hypoglycosylation and Walker-Warburg syndrome. J Med Genet..

[26] Beltrán-Valero de Bernabé D, Currier S, Steinbrecher A, Celli J, van Beusekom E, van der Zwaag B, Kayserili H, Merlini L, Chitayat D, Dobyns WB (2002). Mutations in the O-mannosyltransferase gene POMT1 give rise to the severe neuronal migration disorder Walker-Warburg syndrome. Am J Hum Genet.

[27] Yang AC, Ng BG, Moore SA, Rush J, Waechter CJ, Raymond KM, Willer T, Campbell KP, Freeze HH, Mehta L (2013). Congenital disorder of glycosylation due to DPM1 mutations presenting with dystroglycanopathy-type congenital muscular dystrophy. Mol Genet Metab..

[28] Raphael AR, Couthouis J, Sakamuri S, Siskind C, Vogel H, Day JW, Gitler AD (2014). Congenital muscular dystrophy and generalized epilepsy caused by GMPPB mutations. Brain Res..

[29] Lefeber DJ, Schönberger J, Morava E, Guillard M, Huyben KM, Verrijp K, Grafakou O, Evangeliou A, Preijers FW, Manta P (2009). Deficiency of Dol-P-Man synthase subunit DPM3 bridges the congenital disorders of glycosylation with the dystroglycanopathies. Am J Hum Genet..

[30] Lefeber DJ, de Brouwer APM, Morava E, Riemersma M, Schuurs-Hoeijmakers JHM, Absmanner B, Verrijp K, van den Akker WMR, Huijben K, Steenbergen G (2011). Autosomal recessive dilated cardiomyopathy due to DOLK mutations results from abnormal dystroglycan O-mannosylation. PLoS Genet..

[31] Barone R, Aiello C, Race V, Morava E, Foulquier F, Riemersma M, Passarelli C, Concolino D, Carella M, Santorelli F (2012). DPM2-CDG: a muscular dystrophy—dystroglycanopathy syndrome with severe epilepsy. Ann Neurol..

[32] Longman C, Brockington M, Torelli S, Jimenez-Mallebrera C, Kennedy C, Khalil N, Feng L, Saran RK, Voit T, Merlini L (2003). Mutations in the human LARGE gene cause MDC1D, a novel form of congenital muscular dystrophy with severe mental retardation and abnormal glycosylation of α-dystroglycan. Hum Mol Genet..

[33] Riemersma M, Froese DS, van Tol W, Engelke UF, Kopec J, van Scherpenzeel M, Ashikov A, Krojer T, von Delft F, Tessari M (2015). Human ISPD is a cytidyltransferase required for dystroglycan O-mannosylation. Chem Biol..

[34] Praissman JL, Willer T, Sheikh MO, Toi A, Chitayat D, Lin Y-Y, Lee H, Stalnaker SH, Wang S, Prabhakar PK (2016). The functional O-mannose glycan on α-dystroglycan contains a phospho-ribitol primed for matriglycan addition. Elife..

[35] Kanagawa M, Kobayashi K, Tajiri M, Manya H, Kuga A, Yamaguchi Y, Akasaka-Manya K, Furukawa J-I, Mizuno M, Kawakami H (2016). Identification of a post-translational modification with ribitol-phosphate and its defect in muscular dystrophy. Cell Rep..

[36] Boncompain G, Divoux S, Gareil N, de Forges H, Lescure A, Latreche L, Mercanti V, Jollivet F, Raposo G, Perez F (2012). Synchronization of secretory protein traffic in populations of cells. Nat Methods..

[37] Praschberger R, Balint B, Mencacci NE, Hersheson J, Rubio-Agusti I, Kullmann DM, Bettencourt C, Bhatia K, Houlden H (2015). Expanding the phenotype and genetic defects associated with the GOSR2 gene. Mov Disord Clin Pract..

